# Integrated transcriptomic and metabolomic analyses reveal flavonoid and lipid metabolic reprogramming in *Dendrobiumofficinale* during *Colletotrichum fructicola*-induced anthracnose

**DOI:** 10.7717/peerj.20563

**Published:** 2026-01-15

**Authors:** Jun Yang, Xinqiao Zhan, Yahui Zhang, Yichun Qian, Minxia Pang, Guoxiang Yao, Bizeng Mao

**Affiliations:** 1 Institute of Biotechnology, Ministry of Agriculture Key Lab of Molecular Biology of Crop Pathogens and Insects, Zhejiang Key Laboratory of Biology and Ecological Regulation of Crop Pathogens and Insects, Zhejiang University, Hangzhou, China; 2 School of Pharmaceutical Sciences, Taizhou University, Taizhou, China; 3 Zhejiang Jianjiuhe Group Co., Ltd, Ningbo, China

**Keywords:** Medicinal orchid, Host-pathogen interaction, Secondary metabolite biosynthesis, Fatty acid catabolism

## Abstract

Anthracnose disease caused by *Colletotrichum fructicola* severely compromises the medicinal value and yield of *Dendrobium officinale*. To elucidate the host metabolic response to pathogen infection, we integrated transcriptomic and metabolomic analyses of *D. officinale* challenged with *C. fructicola*. Our results revealed a profound metabolic reprogramming orchestrated by the pathogen, characterized by upregulated flavonoid biosynthesis (*e.g.*, *DFR*, *LDOX* activation) and enhanced lipid catabolism (*e.g.*, β-oxidation *via LACS*, *DECR*, *HACL*). Metabolite profiling demonstrated a significant reduction in phosphatidylcholine (PC) and phosphatidylethanolamine (PE) alongside increased free fatty acids, indicating active lipid degradation. Notably, acyl-CoA intermediates linked lipid catabolism to flavonoid production, suggesting a metabolic axis where pathogen-induced lipid breakdown fuels defense-related secondary metabolite synthesis. This study identifies flavonoid and lipid metabolic reprogramming as critical axes in host-pathogen interactions, providing a foundation for developing targeted disease control strategies.

## Introduction

*Dendrobium officinale* was initially documented in Shen Nong’s Herbal Classic, the earliest extant work on traditional Chinese medicine. It is credited with the traditional functions of nourishing yin and fortifying the stomach. The stem of this plant serves as the medicinal part, commonly referred to as ‘*Fengdou*’, which abounds in polysaccharides and ethanol-soluble extracts possessing bioactive properties (Zhang et al., 2016). These bioactive constituents have been demonstrated to exhibit antioxidant, anti-cancer, and immunomodulatory functions (Wei et al., 2018). Owing to self-sterility, a protracted growth cycle, and excessive exploitation, the wild germplasm resources of *D. officinale* are under threat of endangerment. As a result, artificial cultivation has become the principal source. In China, *D. officinale* is predominantly cultivated in several provinces, including Zhejiang, Yunnan, and Guizhou, with its market value ranging from $450 to $3,100 per kilogram. Nevertheless, the plantation industry of *D*. *officinale* is menaced by numerous fungal pathogens. Prominent among them are *Colletotorichum gloeosporioides* s.s, *C. fructicola*, *Epicoccum sorghinum* (Mirghasempour et al., 2022). *C. fructicola* exhibits a broad host range and strong pathogenicity: in addition to causing anthracnose in *D. officinale* (as documented in Anhui Province Ma, Wang & Guo, 2019), it can also infect diverse industrial crops including papaya, prickly ash, and walnut, leading to serious anthracnose outbreaks (Marquez-Zequera et al., 2018; Wang et al., 2017; Yang et al., 2024). Despite its established threat to multiple economically important plants, research specifically focusing on its interaction with *D. officinale* is relatively scarce. The initial symptoms of *D. officinale* infected by the pathogen manifest as small, slightly sunken, water-soaked spots that are circular, semi-circular, or irregular in shape. As the lesions expand, the centers turn dark greyish, fringed by dark-brown margins and encircled by a tan halo. In more severe cases, the plants experience defoliation and an overall decline in vigor. Ultimately, both the quality and yield of *D. officinale* are compromised due to anthracnose. Currently, in studies on *D. officinale* pathogens, *C*. *gloeosporioides* s.s is the most frequently reported, while reports regarding *C. fructicola* are relatively scarce. Existing research efforts have predominantly centered on uncovering the interaction mechanisms between pathogens and plants, as well as exploring potential research directions for pathogen studies.

Anthracnose disease caused by *C. fructicola* severely impairs the medicinal value and yield of *D. officinale*, a valuable medicinal plant renowned for its bioactive secondary metabolites. Previous studies have indicated that metabolites of the phenylpropanoid and flavonoid pathways in *D. officinale* are enriched following infection by pathogenic fungi such as *Sclerotium delphinii* (Wu et al., 2022). However, scant research has been dedicated to exploring the dynamic regulatory patterns of these biosynthesis pathways, especially flavonoid metabolism, in the specific context of *C. fructicola* infection. Moreover, the role of lipid metabolism, a core component of plant defense responses, in the interaction between *C. fructicola* and *D. officinale* remains largely unclear. Flavonoids constitute a crucial class of secondary metabolites in *D. officinale* (Zhan et al., 2020), exhibiting diverse pharmacological functions including regulating vascular health to prevent sclerosis, and showing great potential in anti-aging, anti-cancer, and immunomodulation (Jin et al., 2016). This makes flavonoid metabolic responses to pathogen invasion a key focus for understanding how *D*. *officinale*’s medicinal quality is affected by *C. fructicola* infection. Lipid metabolism, meanwhile, is closely linked to plant stress resistance *via* processes such as lipid catabolism and the production of signaling molecules, yet its correlation with flavonoid biosynthesis during *C. fructicola* infection has not been systematically explored.

## Materials and Methods

### Fungal isolation and morphological characterization

Fungal isolation and morphological characterization were performed on *D. officinale* leaves exhibiting anthracnose symptoms collected from Wenzhou, Taizhou, Jinhua, and Hangzhou in Zhejiang Province. Samples were surface-sterilized by immersion in 75% ethanol for 30 s, followed by rinsing with sterile water three times, treatment with 0.1% mercuric chloride for 1 min with continuous shaking, and final rinses with sterile water three times. Sterilized tissues (5 mm × 5 mm) were excised from the junction of healthy and diseased areas and placed on potato dextrose agar (PDA) plates, which were incubated at 25 °C in the dark for 5 days. Pure cultures were obtained *via* monospore isolation. Conidial morphology was observed under a microscope, revealing colorless to brown conidiophores producing single-celled, oblong conidia.

Genomic DNA was extracted using a Plant Genomic DNA kit. For molecular identification, fragments of multiple phylogenetic markers were amplified by polymerase chain reaction (PCR). These included the internal transcribed spacer (ITS) region, *β*-tubulin (TUB), glyceraldehyde-3-phosphate dehydrogenase (GAPDH), actin (ACT), calmodulin (CAL), and chitin synthase 1 (CHS-1) genes (Zhang, Li & Zhu, 2022). The primers used for amplification are listed in Table S3.

The PCR reaction mixture (25 µL) consisted of 2.5 µL of 10× PCR buffer with MgCl_2_, 0.5 µL of dNTPs, 0.5 µL of Taq DNA polymerase, 0.5 µL of each forward and reverse primer, 0.5 µL of genomic DNA template, and 20 µL of ddH_2_O. The same thermal cycling protocol was used for the amplification of all gene fragments: initial denaturation at 94 °C for 5 min; followed by 29 cycles of denaturation at 94 °C for 1 min, annealing at 50 °C for 40 s, and extension at 72 °C for 1 min; with a final extension at 72 °C for 10 min.

The amplified products were sequenced, and similarity analysis was conducted using NCBI BLAST. Multiple sequence alignments and a Maximum Likelihood phylogenetic tree were constructed in MEGA 11.0 with 1000 bootstrap replicates based on the concatenated dataset (ITS, TUB, GAPDH, ACT, CAL, CHS-1). The sequences of *C. fructicola* used for phylogenetic analysis are listed in Table S4.

### Pathogenicity test

To fulfill Koch’s postulates, pathogenicity tests were conducted on healthy, one-year-old *D. officinale* plants. A conidial suspension (1 × 10^6^ spores/mL) of the representative *C. fructicola* isolate was prepared from a 7-day-old culture grown on PDA. Leaves were gently wounded with a sterile needle and inoculated by placing a 10 µL droplet of the suspension on each wound. Control plants were treated with sterile distilled water. All plants were covered with plastic bags to maintain high humidity and incubated in a greenhouse at 25 ± 2 °C. Symptoms typical of anthracnose, such as dark brown, sunken lesions, developed on inoculated leaves within 7n each wound. Control plants were treated with sterile distilled water. All plants were covered with plastic bag *C. fructicola* based on morphological and molecular characteristics, thereby fulfilling Koch’ s postulates.

### Soluble sugar, reactive oxygen species, and phospholipase activity analysis

For the detection of soluble sugar content, frozen leaf samples (100 mg) were ground in liquid nitrogen and extracted with one mL of 50% ethanol at 90 °C for 30 min. After centrifugation at 12,000× g for 15 min, the supernatant was collected, and the ethanol was evaporated under vacuum. The residue was dissolved in distilled water, and the soluble sugar content was determined using the anthrone-sulfuric acid method. Briefly, 0.5 mL of the sample solution was mixed with two mL of anthrone reagent (0.2% anthrone in concentrated sulfuric acid), heated at 100 °C for 5 min, and the absorbance was measured at 620 nm using a spectrophotometer. Glucose was used as a standard to construct a calibration curve, and results were expressed as mg glucose equivalents per gram fresh weight.

For reactive oxygen species (ROS) detection using the nitroblue tetrazolium (NBT), infected and control leaf samples (50 mg) were excised and incubated in 0.1 mM NBT solution for 10 min in the dark at 25 °C. Following incubation, leaf samples were dipped in 95% ethanol and heated at 80 °C for 5 min to remove chlorophyll. After cooling to room temperature, the decolorized discs were photographed to visualize blue formazan deposits indicative of superoxide anion (O_2_^•−^) accumulation. For quantitative analysis, the formazan product was extracted with two mL of dimethyl sulfoxide by shaking at 37 °C for 30 min. The extract was centrifuged at 10,000× g for 10 min, and the absorbance of the supernatant was measured at 700 nm using a spectrophotometer.

Phospholipase (PLA) activity was assayed using egg yolk lecithin as a substrate. Leaf samples (200 mg) were ground in liquid nitrogen and extracted with two mL of ice-cold 50 mM Tris–HCl buffer (pH 7.5) containing one mM EDTA and 1% polyvinylpyrrolidone. The extract was centrifuged at 10,000× g for 10 min at 4 °C, and the supernatant was used as the enzyme source. The reaction mixture (one mL) contained 50 mM Tris–HCl (pH 7.5), 5 mM CaCl_2_, 1% (w/v) egg lecithin, and 100 µL of enzyme extract. After incubation at 37 °C for 30 min, the reaction was terminated by adding two mL of chloroform/methanol (2:1, v/v). The mixture was vortexed and centrifuged at 3,000× g for 10 min, and the organic phase was collected. The released fatty acids were quantified by measuring the absorbance at 234 nm after reacting with copper sulfate and 2,2’-bipyridyl. One unit of PLA activity was defined as the amount of enzyme releasing 1 µmol of fatty acid per minute at 37 °C.

### Transcriptome analysis

Total RNA was extracted from 100 mg of frozen infected leaves using the TRIzol reagent (Invitrogen) according to the manufacturer’s protocol. Methods for RNA quantity, cDNA library construction, and transcriptomic analysis were performed according to our previous work (Zhan et al., 2022a). Briefly, RNA quality and quantity were assessed using a NanoDrop 2000 spectrophotometer. Libraries were sequenced on an Illumina HiSeq platform using 150 bp paired-end reads. Raw reads were filtered to remove adaptor sequences, low-quality reads (*Q*-score <20), and reads with >10% unknown nucleotides using fastp. Subsequently, the clean reads were mapped to the reference genome of *D. officinale* (Zhang et al., 2016). Differential gene expression analysis was then carried out using DESeq2 (version 1.30.1), with the criteria set as —log_2_(fold change)— ≥ 1 and adjusted *p* - value <0.05. The raw of RNA-seq data of *C. fructicola* infected plants has been submitted to the BIG Data Center of the Chinese Academy of Sciences (https://bigd.big.ac.cn) with accession number CRA002691 (Zhan, Qian & Mao, 2022).

### Metabolome analysis

The infection leaves were ground into a fine powder in liquid nitrogen from two treatment groups (CK and IN). The methods of metabolites extraction, LC-MS/MS detection and metabolomics analysis were performed according to our previous study (Zhan et al., 2020). Briefly, Metabolite extraction was carried out by using a methanol:water mixture (50:50, volume/volume). The resultant extracts were then analyzed *via* an Orbitrap Exploris 240 Mass Spectrometer (manufactured by Thermo Fisher Scientific), which was coupled with a Vanquish Horizon UHPLC system (also from Thermo Fisher Scientific). The mobile phase consisted of 0.1% formic acid in water (A) and 0.1% formic acid in acetonitrile (B), with a gradient program: 5% B (0–1 min), 5%–95% B (1–14 min), 95% B (14–16 min), 95%–5% B (16–16.1 min), and 5% B (16.1–20 min) at a flow rate of 0.3 mL/min. The mass spectrometer operated in both positive and negative ion modes with a spray voltage of 3.2 kV (positive) and 2.8 kV (negative), capillary temperature of 320 °C, and sheath gas flow rate of 35 arb. Full-scan MS spectra were acquired from m/z 100–1,500 at a resolution of 60,000, and data-dependent MS/MS scans were performed at 15,000 resolution.

Raw data were processed using Thermo Fisher Scientific Compound Discoverer 3.4, including peak detection, alignment, and annotation. Differential metabolite analysis was conducted *via* partial least squares discriminant analysis (PLS-DA) using the R, with variables having variable importance in projection (VIP) >1 and *p*-value <0.05 considered significant. Metabolites were identified by matching accurate masses and MS/MS spectra against the self-established database and the Kyoto Encyclopedia of Genes and Genomes (KEGG). Hierarchical clustering and principal component analysis (PCA) were performed using the R packages pheatmap and FactoMineR to visualize metabolic profiles.

### Statistical analysis

The data are displayed as means ± standard deviations (SD). Data set were treated by hierarchical clustering using the R package pheatmap (v1.0.12) and by PCA using the R package FactoMineR (v2.6). Gene enrichment analysis was used in the R package clusterProfiler (v4.2.2) (Yu et al., 2012).

## Results

### Pathogen isolation and identification

**Figure 1:**
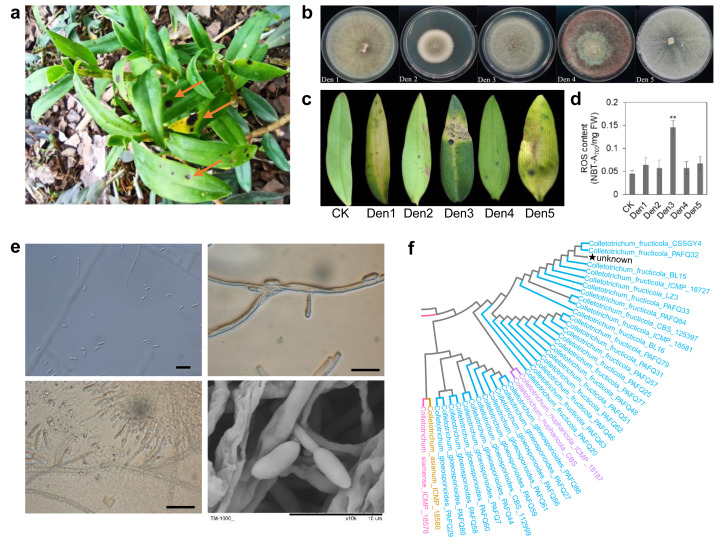
Pathogen isolations and morphological identification. (A) Natural symptoms on leaves of *D. officinale*. (B) Morphological characteristics of five strains from infected leaves. (C) Artificial symptoms on leaves using five strains. Den3 exhibited the same lesions in leaves. (D) ROS content of infected leaves. Values are the mean ± SD (*n* = 10). Student’s *t* test; **p < 0.01. (E) Den3 morphological features under microscope and SEM. Scale bar = 20 µm. (F) Partial phylogenetic analysis of the combined *ITS*, *tub2*, *GAPDH*, *ACT*, *CAL*, and *CHS*-1 sequences using the maximum likelihood method. Bootstrap values are shown on the branches. The pentagram highlights strain Den3.

The symptoms of anthracnose on *D. officinale* were initially observed in Wenzhou, Zhejiang Province. The disease manifests as black spots on leaves, which progress to dark brown lesions with irregular margins and a tan halo (Fig. 1A). To identify the causal pathogen, infected leaf tissues were surface-sterilized, and then incubated on potato dextrose agar (PDA) at 25 °C for 5 days under dark conditions. Five morphotypic groups (Den1–Den5) were obtained and purified through single-spore isolation, based on colony characteristics (size, color, mycelial morphology), edge traits, and spore morphology (Fig. 1B). To confirm the pathogenicity of these five groups and identify the causal agent, pathogenicity tests strictly following Koch’s postulates were conducted. For each group (Den1–Den5), conidial suspensions (1 × 10^5^ conidia/ml) were prepared and inoculated onto the leaves of one-year-old *D. officinale* seedlings without causing mechanical injury; sterile water was used as the control. Each treatment included six biological replicates. All inoculated plants were covered with plastic bags to maintain high humidity and placed in a greenhouse at 25 °C with a 12 h photoperiod. At 25 days post-inoculation (dpi), only seedlings inoculated with the Den3 group exhibited lesions consistent with the field symptoms, including leaf discoloration and wilting (Fig. 1C). In contrast, seedlings inoculated with Den1, Den2, Den4, Den5, and the control group remained asymptomatic. To confirm that all strains within Den3 were pathogenic and conspecific, six isolates were randomly selected from the 87 Den3 strains for supplementary pathogenicity testing (following the same protocol as above, six biological replicates per isolate). All six tested Den3 isolates induced identical typical anthracnose symptoms at 25 dpi, confirming the pathogenicity of Den3 strains as a whole (Fig. S1).

For Koch’s postulate validation, pathogen re-isolation was performed from the lesions of Den3-inoculated seedlings. The re-isolated strains showed identical phenotypic characteristics to the original Den3 group. Microscopic examination of the Den3 group showed conidiophores ranging from colorless to brown, producing single-celled, oblong conidia (Fig. 1E). Molecular identification of both the original and re-isolated Den3 strain was conducted using internal transcribed spacer (ITS), *β*-tubulin, glyceraldehyde-3-phosphate dehydrogenase (GAPDH), actin (ACT), calmodulin (CAL), and chitin synthase 1 (CHS1) gene sequences; the results of the amplification, sequencing, and homology analysis of these markers revealed that the isolate is *C. fructicola* (Fig. 1F; Fig. S1). No fungi were recovered from the leaves of control seedlings. Additionally, quantitative analysis revealed that the Den3-inoculated sites had significantly elevated ROS levels compared to the control tissues (Fig. 1D). Together, these findings confirm *C. fructicola* as the causative agent of anthracnose in *D. officinale* and establish the foundation for subsequent transcriptomic and metabolomic analyses of host-pathogen interactions.

### Transcriptome and metabolome analysis

**Figure 2:**
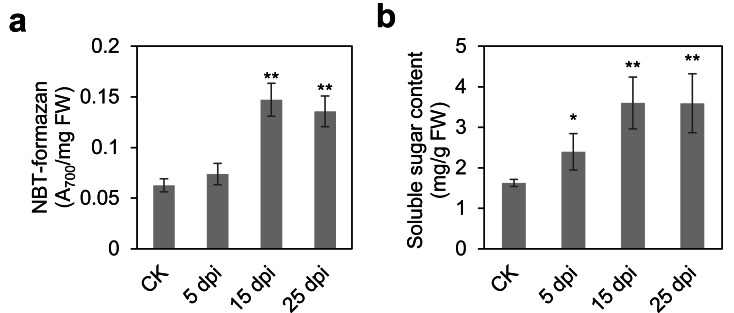
Time course of leaf responses to infection Den3. (A) ROS content were measured at 5, 15, 25 days post-inoculation. (B) Soluble content were measured at 5, 15, 25 days post-inoculation. Values are the mean ± SD (*n* = 10). Student’s *t* test; *p < 0.05, **p < 0.01.

**Figure 3:**
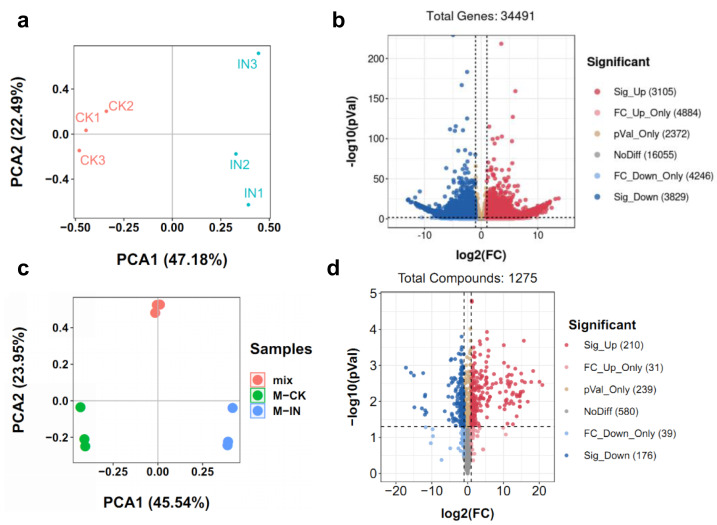
Identification of the DEGs and DAMs between CK and IN. (A) PCA analysis of the transcriptome in CK and IN. (B) Volcano plot showing the significatnt expressed genes in IN *vs.* CK. (C) PCA analysis of the metabolome in CK and IN. (D) Volcano plot showing the significant accumulated metabolites in IN *vs*. CK.

To comprehensively elucidate the molecular and metabolic responses of *D. officinale* to *C. fructicola* infection, transcriptome and metabolome analyses were performed on infected and control leaves at 15 dpi. At this time point, significant increases in ROS and soluble sugar content were observed in infected tissues (Fig. 2), indicating a robust physiological response to pathogen invasion. RNA sequencing yielded high-quality clean reads ranging from 20.13 Gb to 21.89 Gb across all samples (Table S1). PCA revealed a clear separation between control (CK) and infected (IN) groups, with the first two principal components (PC1 and PC2) accounting for 47.18% and 22.49% of the total variance, respectively (Fig. 3A). Differential gene expression analysis identified 6,934 differentially expressed genes (DEGs) in IN *versus* CK, including 3,105 significantly upregulated and 3,829 significantly downregulated genes (Fig. 3B).

Moreover, a total of 1,275 metabolites were detected by nontargeted metabolomics analysis (Table S2). The largest number of metabolites were classified into flavonoids, phenolic acids, and lipids (Fig. S2). PCA of metabolomic data further confirmed distinct metabolic profiles between CK and IN groups, with PC1 and PC2 explaining 45.54% and 23.95% of the variance, respectively (Fig. 3C). PLS-DA identified 210 significantly increased and 176 significantly decreased metabolites in IN *versus* CK, based on VIP >1 and *p*-value <0.05 (Fig. 3D).

### KEGG pathway enrichment analysis of the transcriptome and metabolome

**Figure 4:**
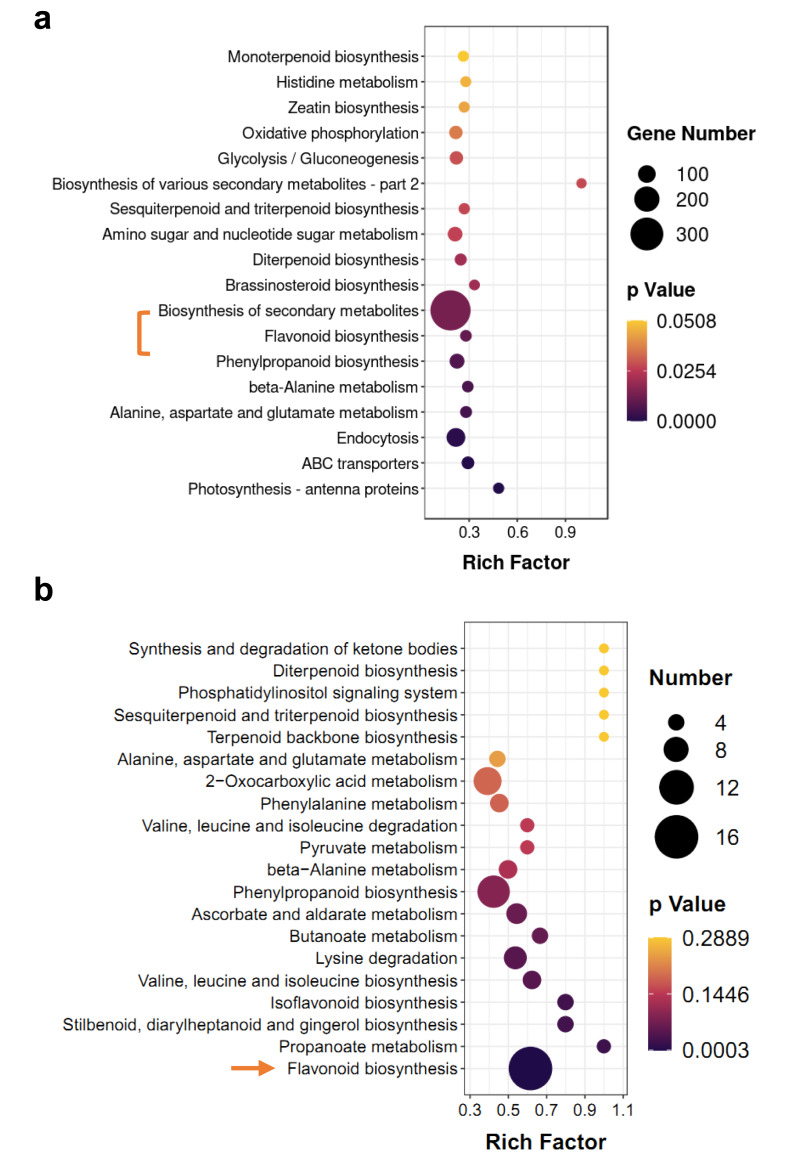
KEGG pathway enrichment analysis of DEGs and DAMs. (A) KEGG enrichment analysis of the DEGs in IN *vs.* CK. (B) KEGG enrichment analysis of the DAMs in IN *vs.* CK. The bar indicates the significant *p*-value.

KEGG enrichment analysis of DEGs and differentially accumulated metabolites (DAMs) showed some of the same pathways, such as ‘flavonoid biosynthesis’ and ‘diterpenoid biosynthesis’ (Fig. 4). However, terpenoid metabolism was non-significant pathway in enrichment analyses, the *p*-values of most of them were greater than 0.05 (Figs. 4A and 4B). Based on hierarchical clustering, DEGs and DAMs of the ‘flavonoid biosynthesis’ pathway were divided into two clusters (Figs. S2 and S3). Among them, two *dihydroflavonol-4-reductase* (*DFR*) genes significantly increased in M-IN *vs.* M-CK, including *LOC110101655* (16.1-fold) and *LOC110111528* (14.8-fold). *Leucoanthocyanidin dioxygenase* (*LDOX*) genes were upregulated 9.2-fold in M-IN *vs.* M-CK (Fig. S3). Eleven flavonoids were significantly increased and five glycosylated flavonoid derivatives were identified in the M-IN group (Fig. S4). These results suggest that flavonoid biosynthesis pathway plays important roles in host-pathogen interactions.

### Anthracnose affects products of lipid turnover

**Figure 5:**
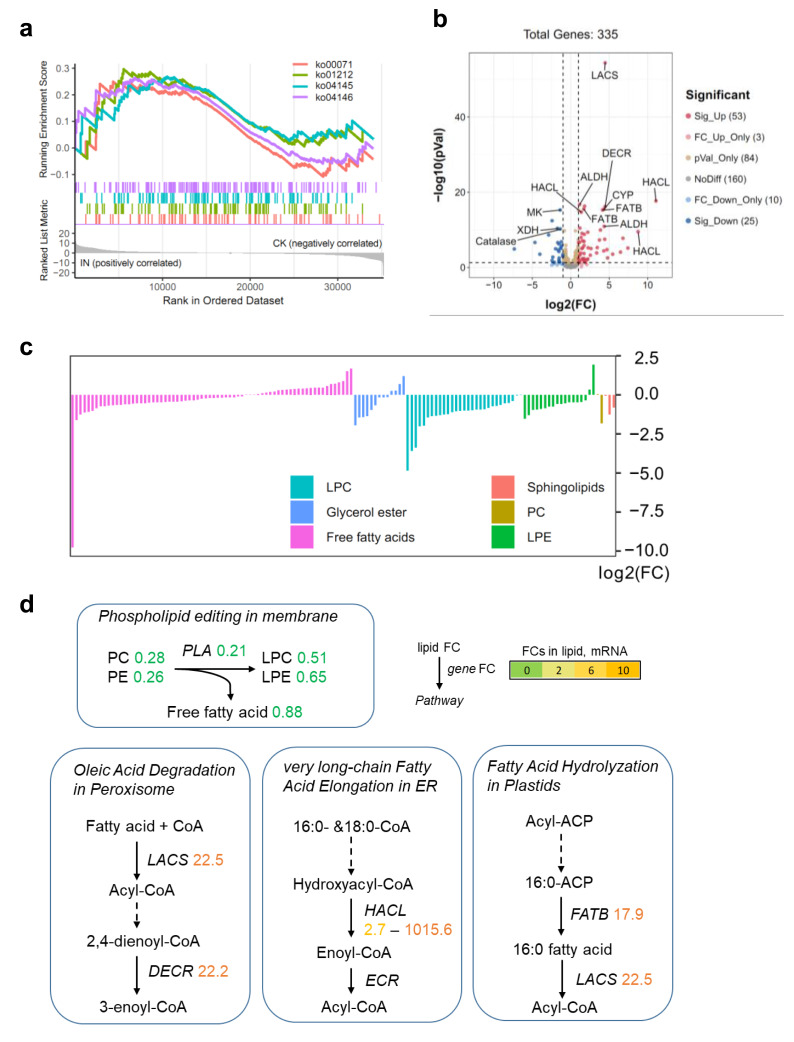
Lipid metabolism shift during *C. fructicola* invasion. (A) GSEA in IN *vs.* CK. Annotations: ko00071, Fatty acid degradation; ko01212, Fatty acid metabolism; ko04145, Phagosome; ko04146, Peroxisome. (B) Volcano plot showing the significatnt expressed genes from GSEA. (C) The accumulated levels of lipid in IN *vs.* CK. (D) Simplified lipid biosynthesis flow base on DEGs and DAMs. FCs in lipid, mRNA (underlined on right) and protein levels (left) are listed and highlighted by different colors according FC values. Abbreviations: LACS, long-chain acyl-CoA synthetase; DECR, peroxisomal 2,4-dienoyl-CoA reductase; CYP, Cytochrome P450 704C1; HACL, 2-hydroxyacyl-CoA lyase; FATB, fatty acyl-ACP thioesterase B; ALDH, aldehyde dehydrogenase (NAD+); MK, mevalonate kinase; XDH, xanthine dehydrogenase; ECR, enoyl-CoA reductase.

To investigate the impact of *C. fructicola* infection on lipid metabolism in *D. officinale*, we integrated transcriptomic and metabolomic analyses. Although no lipid-related pathways were significantly enriched in KEGG analysis of DEGs, Gene Set Enrichment Analysis (GSEA) revealed that lipid catabolism pathways were highly active in infected tissues (IN *vs.* CK) (Fig. 5A). This discrepancy highlights the limitations of predefined pathway annotations in capturing subtle but biologically relevant gene set enrichments. Specifically, 53 genes involved in lipid degradation were upregulated, while 25 were downregulated in IN *versus* CK (Fig. 5B). Metabolite profiling revealed significant reductions in PC and PE levels in infected leaves, alongside decreased free fatty acid content compared to control tissues (Fig. 5C). The conversion of PC to LPC was associated with changes in fatty acid pools and lipid signaling pathways (Fig. 5D). Key genes involved in lipid metabolism, including *long-chain acyl-CoA synthetase* (*LACS*) (22.5-fold upregulation), *peroxisomal 2,4-dienoyl-CoA reductase* (*DECR*) (22.2-fold upregulation), and *2*-*hydroxyacyl*-*CoA lyase* (*HACL*) (1,015.6-fold upregulation), were significantly induced in infected tissues. These genes are central to fatty acid activation, *β*-oxidation, and peroxisomal lipid metabolism, indicating an active shift toward lipid catabolism during pathogen invasion. Additionally, the *fatty acyl*-*ACP thioesterase B* (*FATB*) gene, which mediates 16:0 fatty acid hydrolysis, was upregulated 17.9-fold in IN *versus* CK, further supporting the hypothesis that lipid degradation is a key metabolic response to *C. fructicola* infection.

The impact of pathogen infection on the lipid metabolism of the host is extremely complex. To dissect the mechanism underlying lipid turnover during infection, we measured phospholipase activity and gene expression dynamics at 5, 15, and 25 dpi. Phospholipase activity in infected leaves peaked at 15 dpi, showing a 2.1-fold increase compared to healthy controls (Fig. S5A). qPCR analysis of phospholipase genes (PLA) revealed distinct expression patterns between host and pathogen. Host PLA genes were upregulated at five dpi (2.9*-* fold) but declined thereafter (Fig. S5B), while pathogen-derived PLA2 transcripts remained elevated throughout the infection cycle (5–25 dpi) (Fig. S5C). This suggests that the initial surge in phospholipase activity at five dpi is host-driven, potentially as part of a defense response, whereas sustained activity at later stages (15–25 dpi) is primarily mediated by pathogen-secreted enzymes.

## Discussion

*D. officinale* is a well-known commercial herb in China. *D. officinale* has valuable medicinal components, including dendrobium polysaccharides, dendrobines, terpenoids, and flavonoids, which are valued for health care and pharmaceutical purposes (Zhan et al., 2020; Zhan, Qian & Mao, 2022b; He et al., 2017). Its cultivation, however, is threatened by various fungal pathogens. Among these, *C*. *gloeosporioides* s.s is the most frequently reported agent of anthracnose in *D. officinale* . In contrast, reports concerning *C. fructicola* as a pathogen of this medicinal plant are relatively scarce, despite its documented occurrence in Anhui Province (Ma, Wang & Guo, 2019) . Therefore, the interaction mechanisms between *D. officinale* and *C. fructicola* remain largely unexplored. This study provides one of the first integrated transcriptomic and metabolomic analyses of this specific pathosystem, revealing that flavonoid and lipid metabolic pathways are critically involved in the host’s response to *C. fructicola* infection. Recently, we found that *D. officinale* accumulated more secondary metabolites against environmental factors, such as flavonoid accumulation during freezing (Zhan et al., 2022). Flavonoids are widely distributed in plants to respond environmental stresses. Enhanced accumulation of flavonoids improves plant antioxidant activity under biotic and abiotic stresses. For example, anthocyanin overaccumulation mitigates against oxidative and drought damage in Arabidopsis (Nakabayashi et al., 2014). Flavonoid glycosides overaccumulation enhances tolerance to osmotic stress in rice (Zhan et al., 2019). *Podosphaera aphanis* invasion leads to accumulation of proanthocyanidins and upregulation of flavonoid biosynthesis related genes (Feng et al., 2020). Our transcrpitomics and metabolomics data reveal that the flavonoid biosynthesis pathway is obviously affected by *C*. *fructicola* invasion (Fig. 4). Seven flavonoid biosynthesis genes are significantly upregulated in IN *vs.* CK, including *DFR* and *LDOX* (Fig. S2). *DFR* and *LDOX* are key regulator genes in upstream of anthocyanin biosynthesis (Nakabayashi et al., 2014). *DFR* and *LDOX* have been found to participate delphinidin biosynthesis in purple *Dendrobium* (Zhan et al., 2020). However, delphinidin is not found in IN *vs.* CK by the enrichment analysis of DAMs. Naringenin, chalcone, and dihydroquercetin are significantly increased in IN *vs.* CK (Fig. S3). Naringenin and chalcone are important intermediates in flavonoid metabolism. Dihydroquercetin is a key precursor for flavonol and leucocyanidin biosynthesis (Vogt, 2010). Thus, the roles of *DFR* and *LDOX* in *C*. *fructicola* invasion may be widely related to flavonol and anthocyanin derivatives biosynthesis.

Beyond flavonoid biosynthesis, our findings also unravel a critical role of lipid metabolism reprogramming in host-pathogen interaction. Cultivation of *D. officinale* in greenhouses is hampered by pathogens, including *Sclerotium delphinii*, *Colletotrichum* species, and *Fusarium spp.* (Mirghasempour et al., 2022; Li et al., 2021; Sarsaiya et al., 2020). Recent reports have found lipid metabolism and lipid metabolites involved in plant-microbe interactions. Phosphoinositide (PI) and its derivatives (*e.g.*, PI3P) function as conserved signaling molecules in plant-microbe interactions, as seen in *Phytophthora* infection hyphae where PI3P mediates vesicle trafficking (Qin & Wei, 2021). However, no reports have been found on the regulation of lipid metabolism during pathogens invasions in *Dendrobium*. Phospholipids involve lipid remodeling during environmental pressures, including phosphatidic acid that interacts with proteins, accumulation of unsaturated fatty acids and lysophospholipids under freezing, and galactolipids homeostasis under drought stress (Barrero-Sicilia et al., 2017; Li et al., 2008; Liu, Su & Wang, 2013). The transient upregulation of host PLA genes at five dpi likely reflects an early defense response, whereas sustained pathogen-derived PLA2 expression from 15–25 dpi suggests a virulence strategy to hijack host lipid membranes. This temporal dichotomy underscores the dynamic arms race between host defense and pathogen exploitation of lipid metabolism. Future studies directly comparing the disease severity and metabolic consequences of infection by *C. fructicola* and *C. gloeosporioides* s.s on *D. officinale* would be valuable to determine their relative importance and tailor specific control strategies. The downregulation of PC/PE and upregulation of PLA enzymes (Fig. 5D) suggest phospholipid membrane remodeling, potentially providing acyl-CoA substrates for flavonoid biosynthesis. Acyl-CoA, a central metabolite in lipid catabolism, could serve as a carbon source for flavonoid backbone formation, establishing a metabolic network where lipid degradation fuels secondary metabolite production during pathogen invasion (Jiang et al., 2017). Thus, the regulation of lipid metabolism is an important pathway between *C*. *fructicola* and its host.

## Conclusion

This integrated study elucidates a definitive metabolic defense strategy in *D. officinale* against *C. fructicola*, showing that the host’s anthracnose response involves concerted reprogramming of primary and secondary metabolism—fundamentally linking lipid membrane dismantling to flavonoid-based defense reinforcement—while our data support a model where pathogen-induced phospholipid hydrolysis and concomitant peroxisomal *β*-oxidation activation yield acyl-CoA precursors, a metabolic shift that redirects carbon flux from membrane maintenance to flavonoid synthesis (evidenced by pronounced upregulation of DFR and LDOX and accumulation of key flavonoid intermediates); in essence, this sophisticated host adaptation leverages the catabolism of lipids to support flavonoid anabolism, uncovering a key mechanism for *D. officinale*’s immune response by demonstrating that hijacking lipid-derived carbon skeletons is pivotal for orchestrating chemical defenses during biotic stress.

##  Supplemental Information

10.7717/peerj.20563/supp-1 Supplemental Information 1 Supplemental figures

10.7717/peerj.20563/supp-2 Supplemental Information 2 Supplemental tables

10.7717/peerj.20563/supp-3 Supplemental Information 3 Metabolome dataset A total of 1,275 metabolites were detected by nontargeted metabolomics analysis. The largest number of metabolites were classified into flavonoids, phenolic acids, and lipids.

10.7717/peerj.20563/supp-4 Supplemental Information 4 Transcriptome count dataset RNA sequencing yielded high-quality clean reads ranging from 20.13 Gb to 21.89 Gb across all samples.

10.7717/peerj.20563/supp-5 Supplemental Information 5 ROS content and soluble content

10.7717/peerj.20563/supp-6 Supplemental Information 6 The phylogenetic analysis of the combined ITS, tub2, GAPDH, ACT, CAL, and CHS-1 sequences using the maximum likelihood method. Bootstrap values are shown on the branches The pentagram highlights strain Den3.

## Additional Information and Declarations

### Data Availability

The following information was supplied regarding data availability: The Metabolome data are available at the National Center for Bioinformation: OMIX010942. The raw sequence data are available at the National Genomics Data Center (NGDC) GSA: CRA002691.
