# Supplementary material for: Integrated transcriptomic and metabolomic analyses reveal flavonoid and lipid metabolic reprogramming in Dendrobiumofficinale during Colletotrichum fructicola-induced anthracnose
Source: PeerJ. 2026 Jan 15;14:e20563. doi: 10.7717/peerj.20563 (PMC12812277; doi:10.7717/peerj.20563)
Supplement: Supplemental Information 1 [file peerj-14-20563-s001.pptx]

## Slide 1
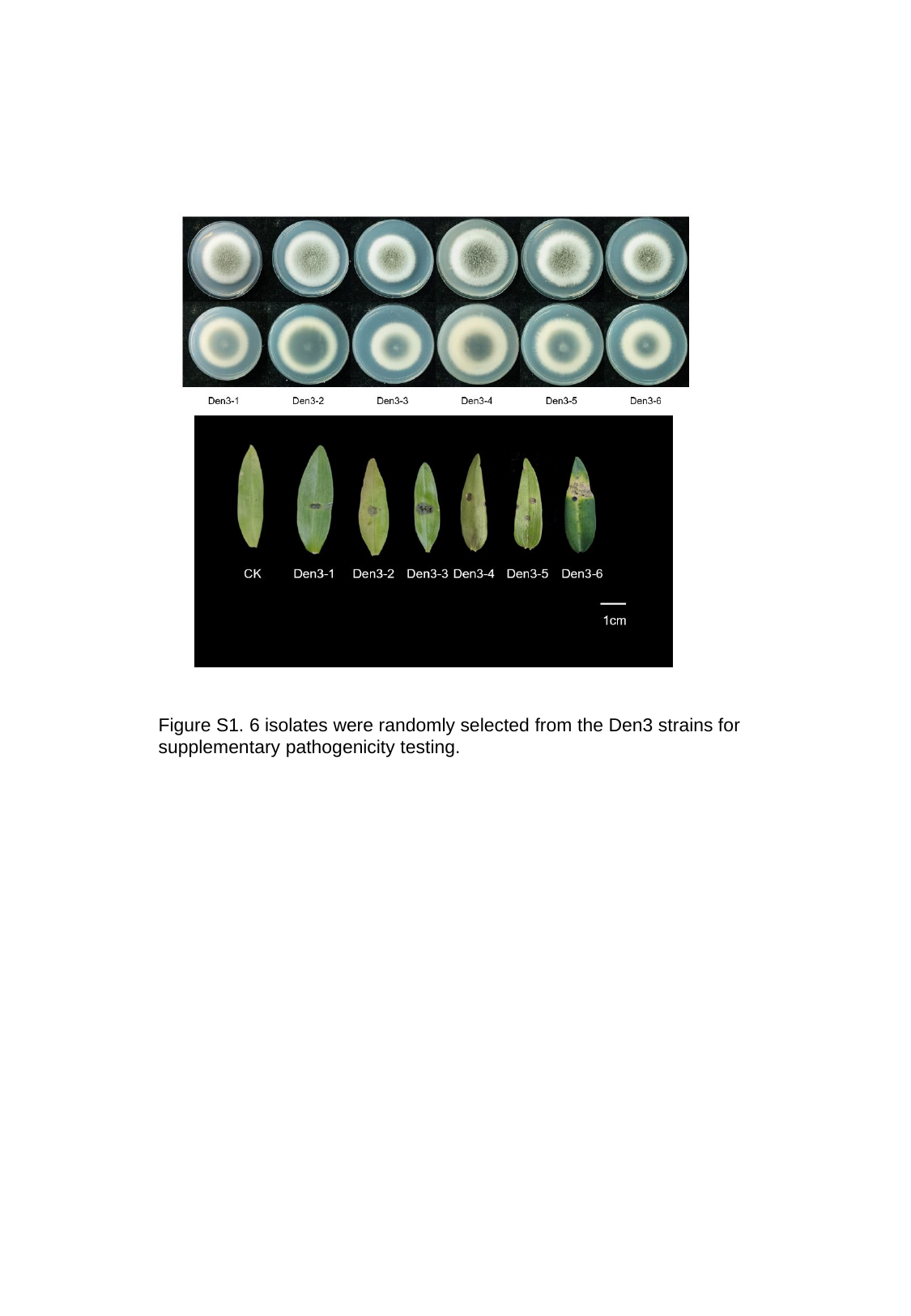

Figure S1. 6 isolates were randomly selected from the Den3 strains for supplementary pathogenicity testing.

## Slide 2
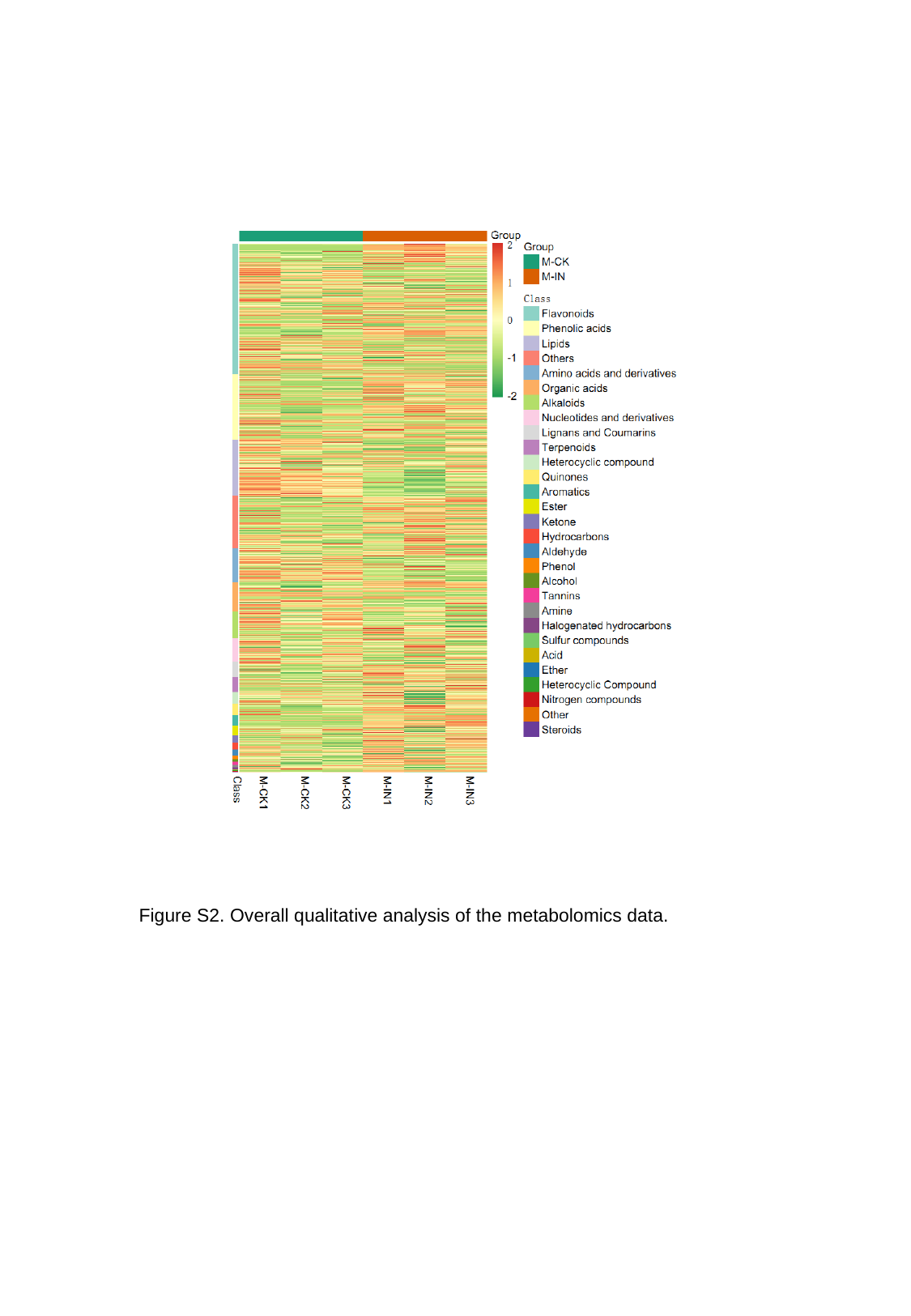

Figure S2. Overall qualitative analysis of the metabolomics data.

## Slide 3
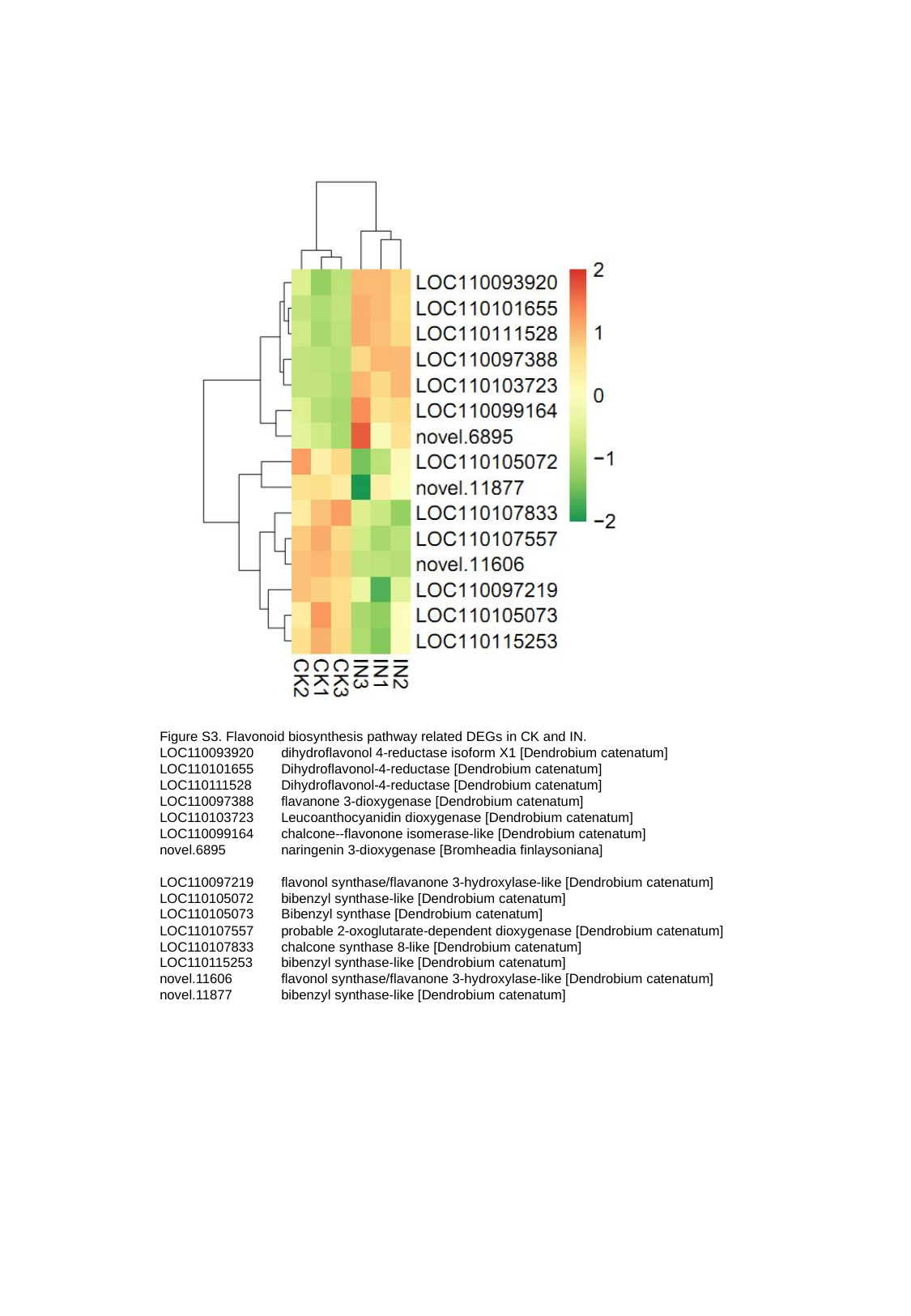

Figure S3. Flavonoid biosynthesis pathway related DEGs in CK and IN.
LOC110093920	dihydroflavonol 4-reductase isoform X1 [Dendrobium catenatum]
LOC110101655	Dihydroflavonol-4-reductase [Dendrobium catenatum]
LOC110111528	Dihydroflavonol-4-reductase [Dendrobium catenatum]
LOC110097388	flavanone 3-dioxygenase [Dendrobium catenatum]
LOC110103723	Leucoanthocyanidin dioxygenase [Dendrobium catenatum]
LOC110099164	chalcone--flavonone isomerase-like [Dendrobium catenatum]
novel.6895	naringenin 3-dioxygenase [Bromheadia finlaysoniana]
LOC110097219	flavonol synthase/flavanone 3-hydroxylase-like [Dendrobium catenatum]
LOC110105072	bibenzyl synthase-like [Dendrobium catenatum]
LOC110105073	Bibenzyl synthase [Dendrobium catenatum]
LOC110107557	probable 2-oxoglutarate-dependent dioxygenase [Dendrobium catenatum]
LOC110107833	chalcone synthase 8-like [Dendrobium catenatum]
LOC110115253	bibenzyl synthase-like [Dendrobium catenatum]
novel.11606	flavonol synthase/flavanone 3-hydroxylase-like [Dendrobium catenatum]
novel.11877	bibenzyl synthase-like [Dendrobium catenatum]

## Slide 4
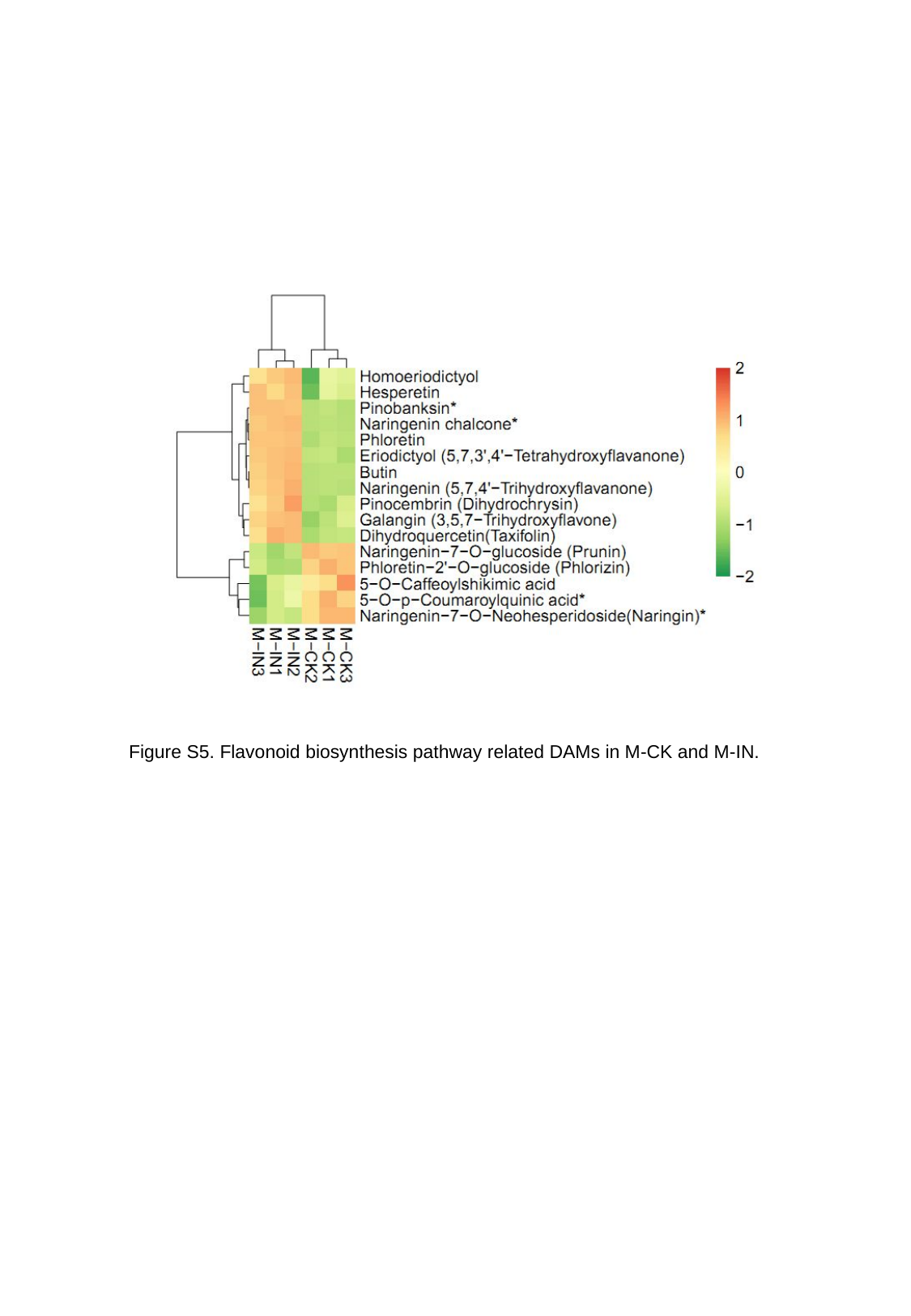

Figure S5. Flavonoid biosynthesis pathway related DAMs in M-CK and M-IN.

## Slide 5
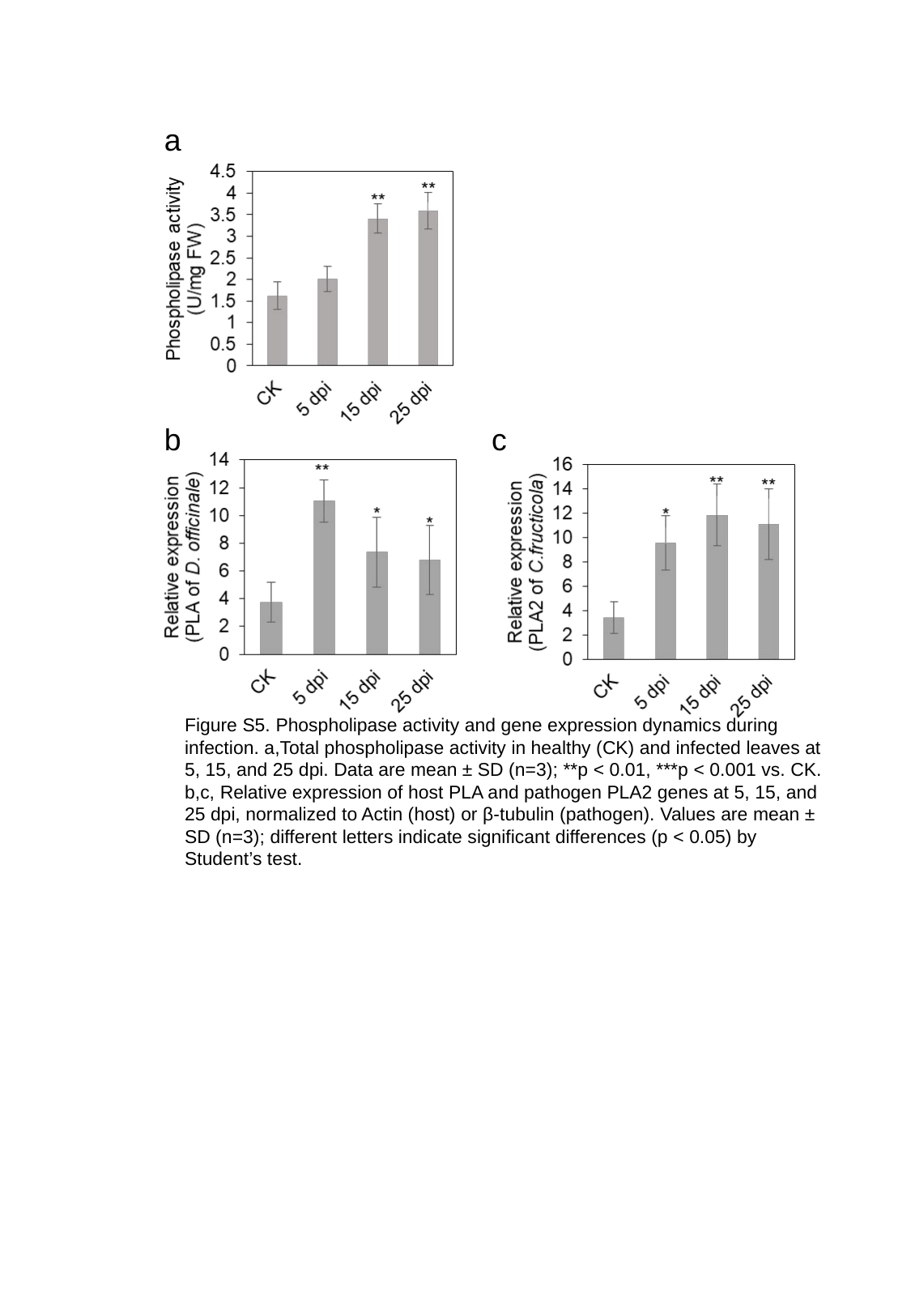

a
b
c
Figure S5. Phospholipase activity and gene expression dynamics during infection. a,Total phospholipase activity in healthy (CK) and infected leaves at 5, 15, and 25 dpi. Data are mean ± SD (n=3); **p < 0.01, ***p < 0.001 vs. CK. b,c, Relative expression of host PLA and pathogen PLA2 genes at 5, 15, and 25 dpi, normalized to Actin (host) or β-tubulin (pathogen). Values are mean ± SD (n=3); different letters indicate significant differences (p < 0.05) by Student’s test.
