# Supplementary figures and images for: Integrated transcriptomic and metabolomic analyses reveal flavonoid and lipid metabolic reprogramming in Dendrobiumofficinale during Colletotrichum fructicola-induced anthracnose
Source: PeerJ. 2026 Jan 15;14:e20563. doi: 10.7717/peerj.20563 (PMC12812277; doi:10.7717/peerj.20563)

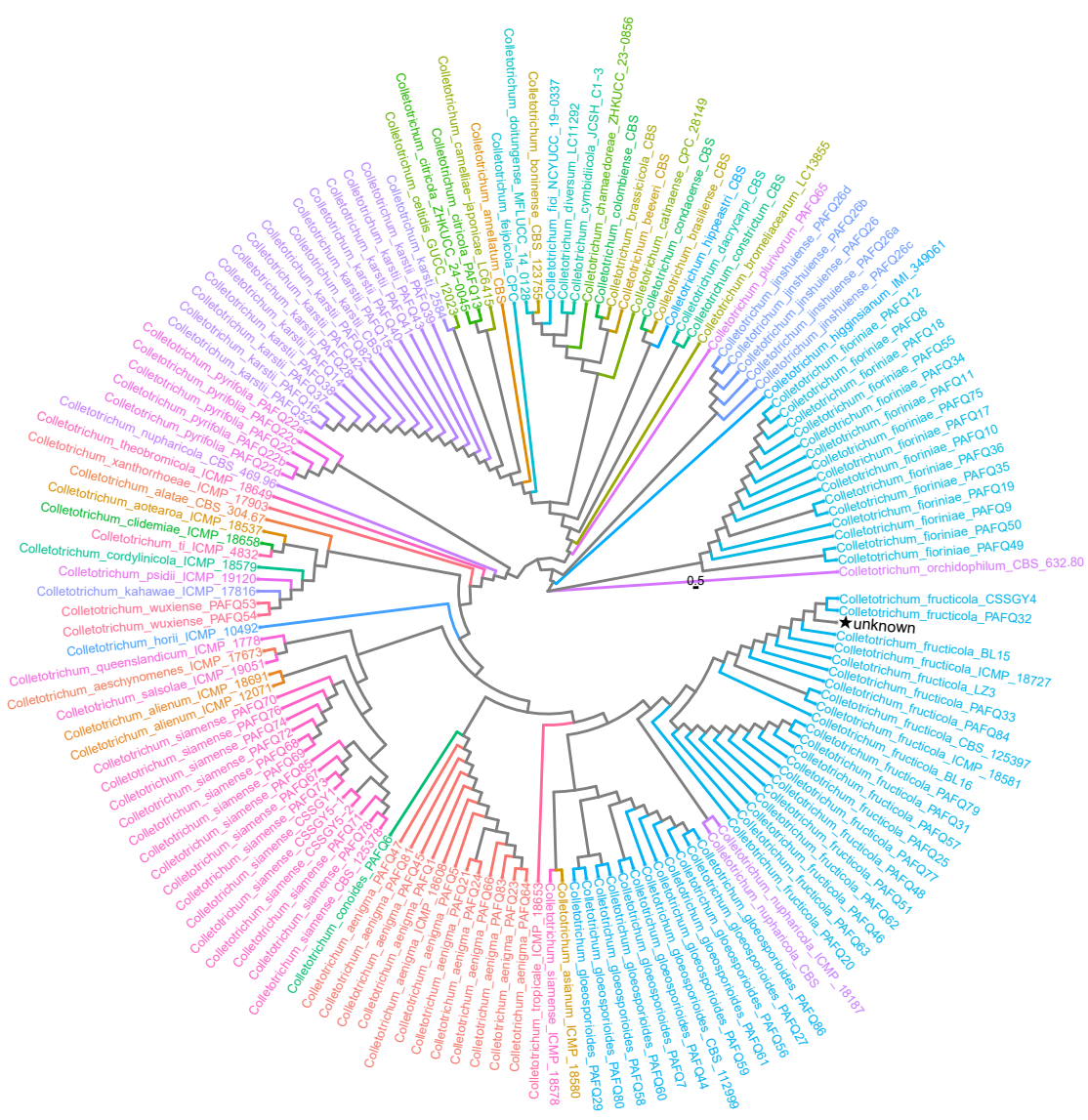

Supplement: Supplemental Information 6 — The pentagram highlights strain Den3. [file peerj-14-20563-s006.pdf]
